# The Components of *Flemingia macrophylla* Attenuate Amyloid **β**-Protein Accumulation by Regulating Amyloid **β**-Protein Metabolic Pathway

**DOI:** 10.1155/2012/795843

**Published:** 2012-06-07

**Authors:** Yun-Lian Lin, Huey-Jen Tsay, Yung-Feng Liao, Mine-Fong Wu, Chuen-Neu Wang, Young-Ji Shiao

**Affiliations:** ^1^Division of Medicinal Chemistry, National Research Institute of Chinese Medicine, Taipei 112, Taiwan; ^2^Institute of Neuroscience, National Yang-Ming University, Taipei 112, Taiwan; ^3^Institute of Biopharmaceutical Science, National Yang-Ming University, Taipei 112, Taiwan; ^4^Division of Basic Chinese Medicine, National Research Institute of Chinese Medicine, Taipei 112, Taiwan

## Abstract

*Flemingia macrophylla* (Leguminosae) is a popular traditional remedy used in Taiwan as anti-inflammatory, promoting blood circulation and antidiabetes agent. Recent study also suggested its neuroprotective activity against Alzheimer's disease. Therefore, the effects of *F. macrophylla* on A**β** production and degradation were studied. The effect of *F. macrophylla* on A**β** metabolism was detected using the cultured mouse neuroblastoma cells N2a transfected with human Swedish mutant APP (swAPP-N2a cells). The effects on A**β** degradation were evaluated on a cell-free system. An ELISA assay was applied to detect the level of A**β**1-40 and A**β**1-42. Western blots assay was employed to measure the levels of soluble amyloid precursor protein and insulin degrading enzyme (IDE). Three fractions of *F. macrophylla* modified A**β** accumulation by both inhibiting **β**-secretase and activating IDE. Three flavonoids modified A**β** accumulation by activating IDE. The activated IDE pool by the flavonoids was distinctly regulated by bacitracin (an IDE inhibitor). Furthermore, flavonoid 94-18-13 also modulates A**β** accumulation by enhancing IDE expression. In conclusion, the components of *F. macrophylla* possess the potential for developing new therapeutic drugs for Alzheimer's disease.

## 1. Introduction

*Flemingia macrophylla *(Leguminosae) is a popular traditional remedy used in Taiwan [1] and India [2]. The stems or leaves have been used as an anti-inflammatory, blood circulation promotion and antidiabetic agent, all of which were relevant to the pathogenesis of Alzheimer's disease (AD). Recent research has suggested its neuroprotective activity against amyloid *β* (A*β*) [3], hepatoprotective activity [4], antiinflammatory activity [5], and antiosteoporosis activity [6].

AD is a complex mental illness characterized by the accumulation of extracellular senile plaques and intracellular neurofibrillary tangles. Senile plaques are composed of deposited A*β*, derived from the processing of amyloid precursor protein (APP) by two enzymes: *β*-site APP cleaving enzyme (BACE or *β*-secretase) and *γ*-secretase [7]. According to the amyloid hypothesis, abnormal accumulation of A*β* in the brain is the primary causative factor contributing to AD pathogenesis, whereby the disease process is believed to result from an imbalance between A*β* production (anabolic activity) and clearance (catabolic activity) [8–10]. APP molecules are cleaved by secretases at the cell surface, the Golgi complex, and along the endosomal/lysosomal pathway [11, 12]. Most cell surface *β*-secretase is reinternalized into early endosomal compartments, from where it can be recycled back to the cell surface or later be redirected to endosomal/lysosomal compartments and/or to the *trans*-Golgi [13]. It is generally believed that removal of A*β* from the brain might be of great therapeutic benefit [14]. Consequently, therapeutic strategies aiming to decrease A*β* levels, such as inhibition of either *β*-secretase or *γ*-secretase and A*β* immunization, are currently a major focus of AD research [15–17]. Much more attention has been paid to abnormal A*β* production, but recently, the role of A*β* degradation in A*β* homoeostasis has been increasingly recognized, as several enzymes that degrade A*β* have been identified, such as insulin degrading enzyme (IDE), neprilysin (NEP), and matrix metalloproteins (MMPs) [18].

Clinical and epidemiological studies have found that type 2 diabetes and hyperinsulinemia increased the risk of developing AD, and the link between these two diseases may be IDE [19, 20]. IDE is a zinc metalloendopeptidase that is highly expressed in the liver, testis, muscle, and brain. Although it is predominantly cytosolic, a secreted form of IDE in extracellular compartments such as cerebrospinal fluid was also identified [21]. IDE degrades a wide range of substrates that include insulin, amylin, insulin-like growth factors, and A*β* [18]. Furthermore, previous work has reported that the IDE level in AD is reduced [22].

In this study, we investigate the effect of *F. macrophylla *extracts or isolated pure compounds on A*β* accumulation and found that they decrease extracellular accumulation of A*β*1-40 in the cultured mouse neuroblastoma cells N2a transfected with human Swedish mutant APP (swAPP-N2a cells) by inhibiting *β*-secretase or enhancing A*β* degradation.

## 2. Methods

### 2.1. Reagents

 Medium for cell culture, heparin, Lipofectamine, and human *β* amyloid 1-40 and 1-42 kits were purchased from Invitrogen (Carlsbad, CA, USA). Mouse anti-actin antibody and anti-IDE polyclonal antibody, rabbit polyclonal anti-APP (KPI domain) antibody, and synthetic A*β*1-40 were purchased from Millipore (Billerica, MA, USA). Anti-A*β*1-17 antibody (clone 6E10) was from Signet (Dedham, MA). Enhanced chemiluminescence detection reagents, anti-rabbit and anti-mouse IgG antibody conjugated with horseradish peroxidase were obtained from GE Healthcare (Buckinghamshire, UK). Insulin, progesterone, putrescine, sodium selenite, and transferrin were purchased from Sigma (St. Louis, MO, USA). All other reagents were purchased from Sigma (St. Louis, MO, USA) or Merck (Darmstadt, Germany).

### 2.2. Plant Material, Extraction, and Isolation

**Figure 1:**
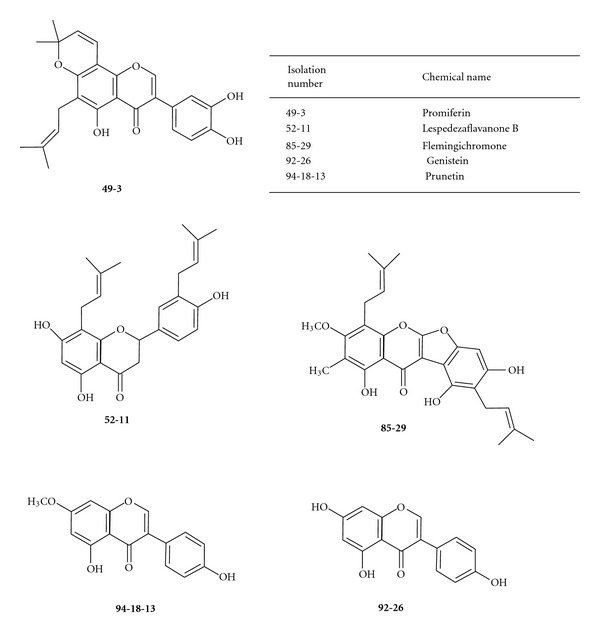
The structure and chemical name of the flavonoids isolated from *F. macrophylla*.

The aerial parts of *F. macrophylla *were collected from Kaohsiung County, Taiwan in May, 2002. The plant was identified by Mr. Jun-Chih Ou, former associate investigator of National Research Institute of Chinese Medicine, and comparison with the voucher specimens was deposited earlier at the Herbarium of the Department of Botany, National Taiwan University, Taipei, Taiwan (no. TAI219262, April, 1988). The extraction and isolation of each fraction for this assay is listed in Table 1, and the structure and chemical name of the flavonoids isolated from *F. macrophylla* were displayed in Figure 1.

### 2.3. Cell Culture and Transfection

Neuro-2a (N2a) cells were cultured in minimal essential medium (MEM) containing 10% fetal bovine serum (FBS). Confluent 90% N2a cells were transfected with plasmid containing human Swedish mutant of amyloid precursor protein (pCGR/APP_770_) by using Lipofectamine 2000. After transfection for 6 h, the cells were incubated with chemical defined medium (DMEM/F12 medium containing 5 mM Hepes pH 7.4, 0.6% glucose, 2.5 mM glutamine, 3 mM NaHCO_3_, 100 *μ*g/mL, transferring, 20 nM progesterone, 60 *μ*M putrescine, 30 nM sodium selenite, 2 *μ*g/mL heparin, and 100 nM insulin) for 20 h. For treatment with cells, the fractions or flavanoids of *F. macrophylla *were introduced into the chemical defined medium.

### 2.4. MTT Assay

The reduction of 3-[4,5-dimethylthiazol-2-yl]-2,5-diphenyl-tetrazolium bromide (MTT) was used to evaluate cell viability. Cells were incubated with 0.5 mg/mL MTT for 1 h. The formazan particles were dissolved with DMSO. OD_600 nm_ was measured using an ELISA reader. 

### 2.5. The Cell-Free Assay of A*β*1-40 Degradation

The conditioned medium of N2a cells containing the proteases to degrade A*β* was collected and used for the cell-free assay of A*β* degradation. Ten ng of synthetic A*β*1-40 (Invitrogen, 03-138) were added into 300 *μ*L N2a-conditioned medium containing various reagents and incubated at 37°C for 24 h. The remaining A*β* were then quantified by ELISA assay kit.

### 2.6. Quantification of A*β*1-40 in Cells and Culture Medium

After treatment, culture media and cell were collected separately and subjected to determining the levels of A*β*1-40 using assay kits. The detailed experiments were performed according to the manufacturer's protocol.

### 2.7. Immunoblotting

After treatment, culture media were collected and cells were washed with ice-cold phosphate buffered saline (PBS) three times. Cells were harvested in lysis buffer (50 mM Hepes pH7.5, 2.5 mM EDTA, 1 mM phenylmethylsulfonyl fluoride, 5 *μ*g/mL aprotinin, and 10 *μ*g/mL leupeptin), and cell lysates were prepared. Equal protein amounts of cell lysate and equal volume of culture medium were subjected to SDS-polyacrylamide gel electrophoresis and immunoblotting. Fujifilm LAS-3000 (Tokyo, Japan) was used to detect and quantify the immunoreactive protein.

### 2.8. Statistical Analysis

Results are expressed as mean ± SD and were analyzed by ANOVA with post hoc multiple comparisons with a Bonferroni test.

## 3. Results

### 3.1. The Effects of Insulin and Bacitracin on A*β*1-40 Level in swAPP-N2a Cells Culture

**Figure 2:**
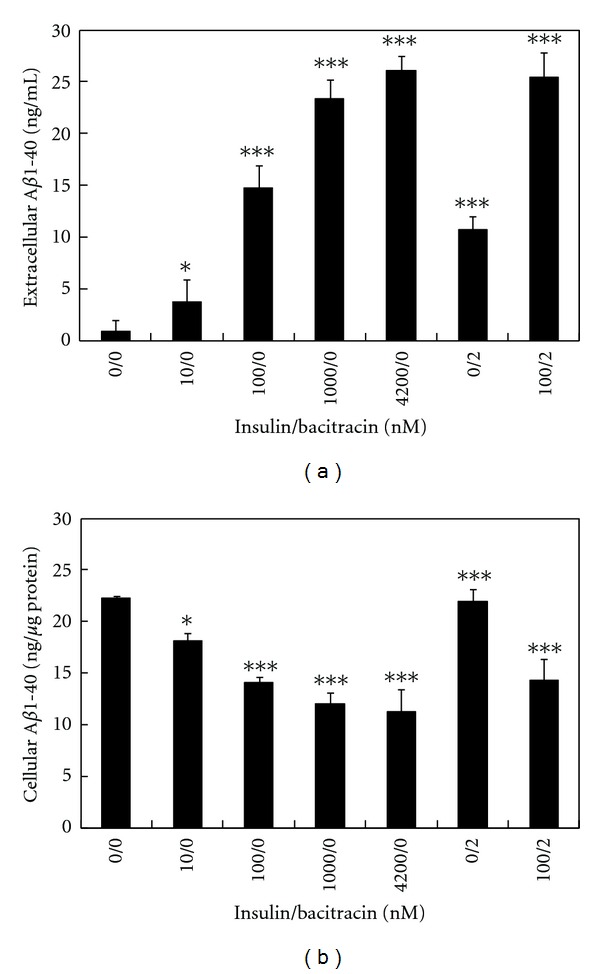
The effect of insulin and bacitracin on the level of extracellular and intracellular A*β*1-40. APP-transfected N2a cells were treated with indicated concentrations of insulin and bacitracin for 20 h. The level of extracellular (a) and intracellular (b) A*β*1-40 was determined by ELISA. Results are means ± SD from three independent experiments. Significant differences between control and treated cells are indicated by *P < 0.05, ***P < 0.001.

To determine the importance of IDE activity on the levels of both extracellular and intracellular A*β*1-40 in swAPP-N2a cell culture, various concentrations of insulin (the substrate of IDE) and/or 2 nM bacitracin (a competitive inhibitor of IDE) were subjected into swAPP-N2a cell culture, and the A*β*1-40 accumulation was assayed. The results showed that insulin promotes A*β*1-40 accumulation in a concentration-dependent manner. Extracellular A*β*1-40 was hardly detected (i.e., 0.88 ± 1.07 ng/mL) in the culture medium without containing insulin. Insulin at 10, 100, 1000, and 4200 nM increased the level of extracellular A*β*1-40 to 3.70 ± 2.18 ng/mL, 14.78 ± 2.17 ng/mL, 23.38 ± 1.83 ng/mL, and 26.02 ± 1.45 ng/mL, respectively (Figure 2(a)). The results suggested that about 26 ng/mL of extracellular A*β*1-40 in the cultured medium regulated by insulin sensitive peptidase(s), including IDE. Therefore, bacitracin was employed to verify the IDE-sensitive pool of extracellular A*β*1-40 in the cultured medium. The results showed that 2 nM bacitracin increased the level of extracellular A*β*1-40 to 10.66 ± 1.32 ng/mL (Figure 2(a)), and higher concentration of bacitracin did not significantly enhance this effect, suggesting that about 11 ng/mL of extracellular A*β*1-40 in the cultured medium was regulated by IDE.

Insulin may regulate the extracellular A*β*1-40 by enhancing exocytosis of the intracellular A*β*1-40. Therefore, the level of intracellular A*β*1-40 was assayed. The results showed that the level of intracellular A*β*1-40 was concentration dependently reduced by insulin, but not by bacitracin (Figure 2(b)). The results suggested that insulin may promote the level of extracellular A*β*1-40 by inhibiting IDE and by accelerating the exocytosis of intracellular A*β*1-40. Alternately, bacitracin did not affect the exocytosis of intracellular A*β*1-40. The similar effects of insulin and bacitracin were found on A*β*1-42 (data not shown).

### 3.2. The Effects of Insulin and Bacitracin on the Degradation of Synthetic A*β*1-40 in the N2a-Conditioned Medium

**Figure 3:**
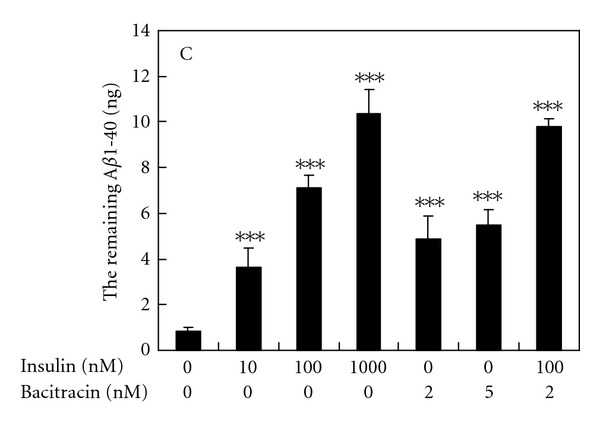
The effect of insulin and bacitracin on A*β*1-40 degradation in the N2a-conditioned medium. A*β*1-40 (10 ng) were incubated in the N2a-conditioned medium with indicated concentrations of insulin and bacitracin, at 37°C for 16 h. The level of remaining A*β*1-40 was determined by ELISA. Results are means ± SD from three independent experiments. Significant differences between control and FM fractions-treated cells are indicated by ***P < 0.001.

For bypassing the involvement of A*β* anabolic and trafficking pathway, a cell-free A*β* degradation assay using N2a cell-conditioned medium as the source of secreted protease and the synthetic A*β*1-40 was employed as the substrate. The results showed that A*β* degradation was inhibited by insulin in a concentration-dependent manner (Figure 3). The added synthetic A*β*1-40 (10 ng) was degraded to 0.83 ± 0.17 ng in the conditioned medium without containing insulin, and 10, 100, and 1000 nM insulin increased the level of remaining A*β*1-40 to 3.65 ± 0.82 ng, 7.13 ± 0.55 ng, and 10.34 ± 1.11 ng, respectively, indicating that the degradation of 10 ng A*β* was completely abolished by 1 *μ*M insulin (Figure 3). Treatment with 2 nM, 5 nM bacitracin, or 2 nM bacitracin combined with 100 nM insulin increased the remaining level of A*β*1-40 to 4.83 ± 0.96, 5.51 ± 0.65, and 9.81 ± 0.35 ng, respectively (Figure 3), suggesting a synergism of insulin and bacitracin on inhibiting A*β* degradation.

### 3.3. The Effects of the Fractions and Flavonoids Isolated from *F. macrophylla* on the Level of A*β*1-40

To determine the effects of the fractions and flavonoids isolated from *F. macrophylla *on the level of extracellular A*β*1-40, the cell toxicity of the fractions and flavonoids was detected, and was then the subtoxic concentration (STC) of the fractions and flavonoids was subjected into the extracellular A*β*1-40 accumulation assay. The results indicated that five highly polar fractions (i.e., EtOH, H_2_O, H75M, B50M, and B75M) attenuated the accumulation of medial A*β*1-40 by more than 50% (Table 2). Among the lesser polar fractions, EA-74 is the most effective fraction which attenuated the accumulation of medial A*β*1-40 to 52.62 ± 12.56% of control (Table 2). Three flavonoids (i.e., 49-2, 52-11, and 94-18-13) attenuated the accumulation of extracellular A*β*1-40 by more than 30% (Table 3).

The effects of the fractions and flavonoids on the intracellular A*β*1-40 accumulation were further evaluated. The result showed that the fraction H_2_O, H75M, and B75M elevated the intracellular level of A*β*1-40 to 155.6 ± 13.4, 213.5 ± 47.3, and 238.8 ± 60.3% of the control, respectively, and the fraction EtOH, B50M, and EA-74 and the flavonoid 49-2, 52-11, and 94-18-13 did not exert significant effects on the intracellular A*β*1-40 accumulation (Tables 2 and 3). Those were therefore selected for further investigation.

### 3.4. The Effects of the Fractions and Flavonoids Isolated from *F. macrophylla* on the A*β*1-40 Degradation in the N2a-Conditioned Medium

**Figure 4:**
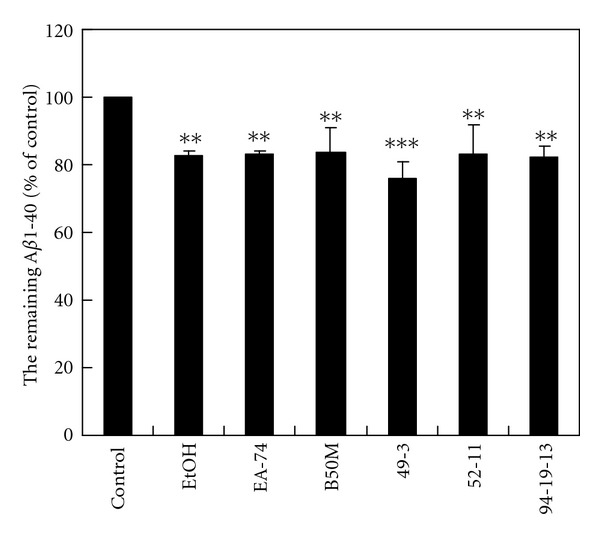
The effects of the fractions and flavonoids of *F. macrophylla* on A*β*1-40 degradation in the N2a-conditioned medium. A*β*1-40 (10 ng) were incubated in the N2a-conditioned medium with 100 nM insulin and the fractions and flavonoids of *F. macrophylla* at NTC, 37°C for 20 h. The level of remaining A*β*1-40 was determined by ELISA. Results are means ± SD from three independent experiments. Significant differences between control and the treated cells are indicated by **P < 0.01, ***P < 0.001.

The fraction EtOH, EA-47, B50M, and flavonoid 49-3, 52-11, and 94-19-13 decreased the remaining synthetic A*β*1-40 to 82.66 ± 1.26%, 83.25 ± 0.74%, 83.50 ± 7.30%, 76.02 ± 4.88%, 83.24 ± 8.60%, and 82.31 ± 8.04% of the control, respectively (Figure 4). The results suggesting that the fractions and flavonoids may ameliorate A*β* accumulation by promoting A*β* degradation. The similar effects were found on A*β*1-42 (data not shown).

### 3.5. The Level of Secreted IDE Was Promoted by Flavonoid 94-18-13

**Figure 5:**
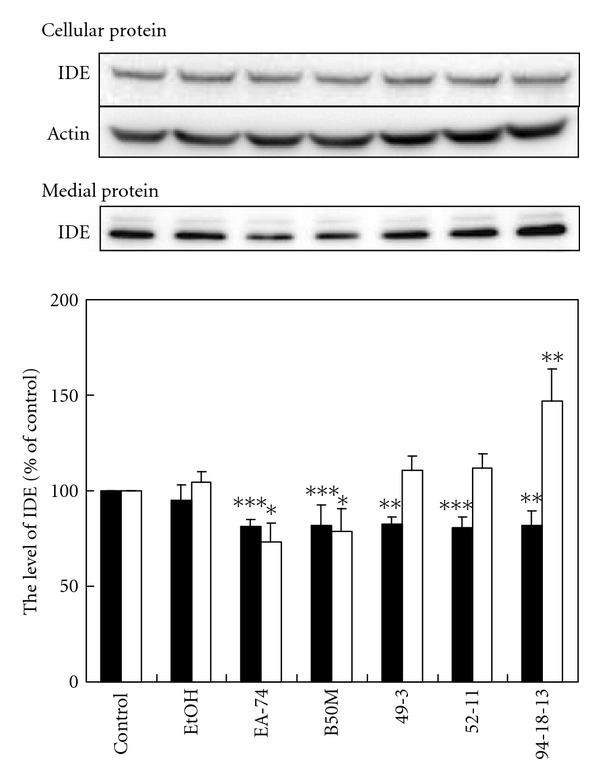
The level of IDE was differentially affected by the fractions and flavonoids of *F. macrophylla*. N2a cells were treated with fractions and flavonoids for 20 h at NTC. The level of IDE in cell lysate and medium was determined by immunoblotting. The upper panel is the representative blot. The lower panel is the relative level of IDE in cell lysate (closed column) and medium (opened column) exhibited as percentage of the control. Results are means ± SD from three independent experiments. Significant differences between control and the treated cells are indicated by *P < 0.05, **P < 0.01 and ***P < 0.001.

Treatment with the fraction B50M, EA-74, and flavonoid 49-2, 52-11, and 94-18-13 attenuated the level of cellular IDE by about 20%, whereas the fraction EtOH failed to show significant effect on the level of cellular IDE (Figure 5). Treatment with the faction B50M and EA-74 eliminated the level of medial IDE by 21.3 ± 12.0 and 26.6 ± 9.9%, respectively. On the contrary, flavonoid 94-18-13 increased the level of medial IDE to 146.9 ± 16.7% of the control. The result suggested that only flavonoid 94-18-13 may accelerate A*β* degradation by promoting IDE expression. The change in enzyme activity may also be involved although it is not detected in this study.

### 3.6. The Recovery Effect of Bacitracin on the Treatment-Reduced Accumulation of Extracellular A*β*1-40

**Figure 6:**
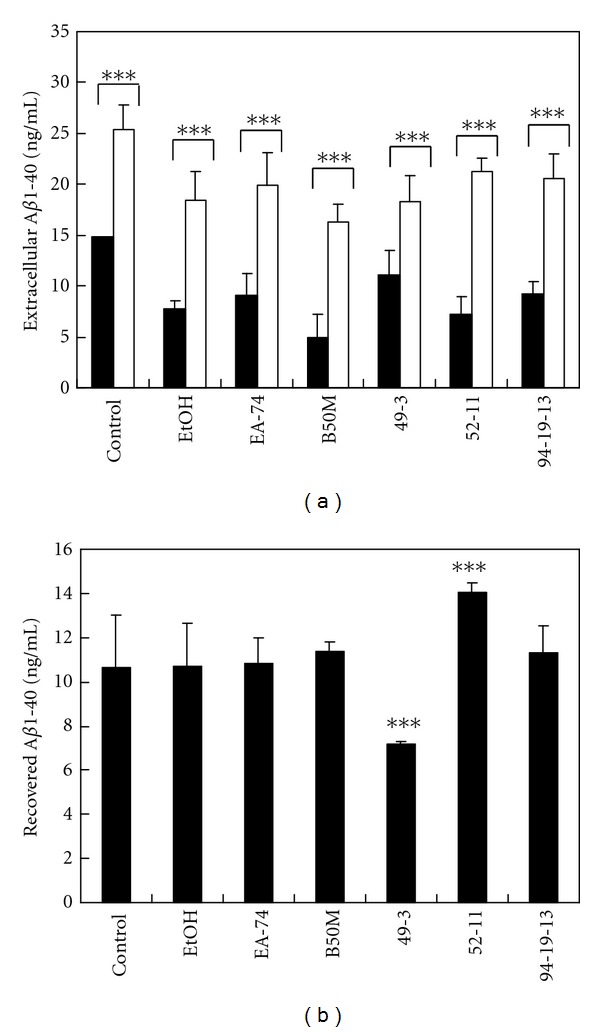
A*β*1-40 accumulation reduced by the fractions and flavonoids was differentially recovered by bacitracin. (a) swAPP_770_-transfected N2a cells were treated with the fractions and flavonoids for 20 h at NTC, in the absence (closed columns) and presence (opened columns) of 2 nM bacitracin. The level of extracellular A*β*1-40 was determined by ELISA. (b) The recovery effect of bacitracin was calculated by the subtraction between the levels of the cells treated with and without bacitracin. Results are means ± SD from three independent experiments. Significant differences between control and FM fractions-treated cells are indicated by ***P < 0.001.

The promoting activity of the fractions and flavonoids on A*β* degradation by IDE may include bacitracin-sensitive and -insensitive pools. The bacitracin-sensitive pools in the cultures treated with the fraction EtOH, EA-74, B50M, and flavonoid 94-19-13 were 10.71, 10.83, 11.35, and 11.29 ng/mL, respectively (Figure 6). The results suggested that these treatments did not affect the bacitracin-sensitive pool. The bacitracin-sensitive pools in the cultures treated with the flavonoid 49-3 and 52-11 were 7.18 and 14.05 ng/mL, suggesting that flavonoid 49-3 and 52-11 reduce and enhance the bacitracin-sensitive pool, respectively.

### 3.7. The Level of Soluble APP*β* Was Decreased by the Fractions of *F. macrophylla*

**Figure 7:**
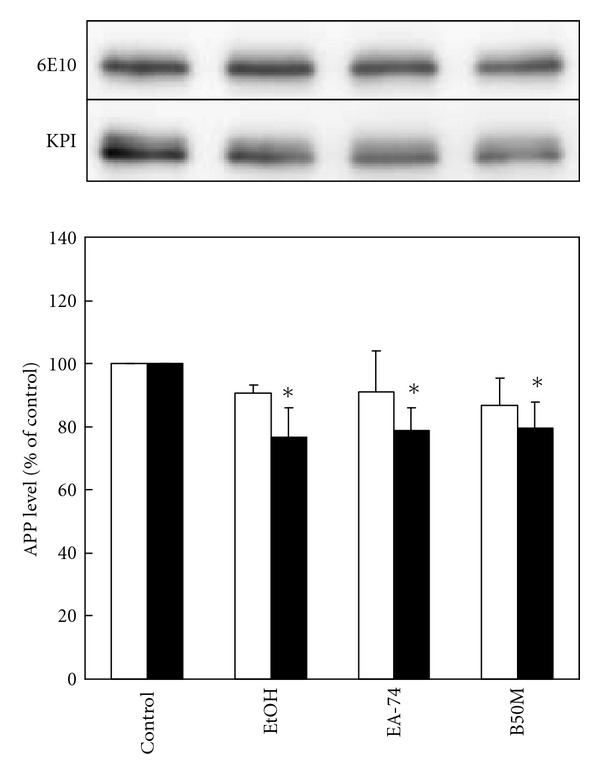
The effect of the fractions of *F. macrophylla* on the level of secreted APP in swAPP770-transfected N2a cell. swAPP_770_-transfected N2a cells were treated with the fractions for 20 h at NTC of 10, 1, and 10 *μ*g/mL, respectively. The level of 6E10 antibody-stained sAPP (i.e., sAPP*α*) and KPI antibody-stained sAPPs (i.e., sAPP*α* plus sAPP*β*) in conditioned medium was determined by immunoblotting. The upper part is the representative image of immunoblot. The lower part is the relative level of 6E10 antibody-stained sAPP*α* (opened column) and KPI antibody-stained sAPPs (closed column). Results are means ± SD from three independent experiments. Significant differences between control and FM fractions-treated cells are indicated by *P < 0.05.

The anabolic pathway of A*β* may also be affected by the fractions of *F. macrophylla* which resulted in the decrease of extracellular A*β*. Two categories of soluble APP (sAPP) including *α*-secretase-derived sAPP (sAPP*α*) and *β*-secretase-derived sAPP (sAPP*β*) may be detected in the swAPP-N2a-conditioned medium. Two antibodies were used to detect these two sAPPs. The anti-A*β*1-17 (6E10) antibody may recognize sAPP*α* (this fragment contains A*β*1-17), and the anti-APP (KPI domain) antibody may recognize both sAPP*α* and sAPP*β* on immunoblot. The result showed that 6E10 antibody-recognized sAPP*α* was not significantly affected by the fractions of *F. macrophylla*. By contrast, the anti-APP (KPI domain) antibody-recognized sAPP*α* and sAPP*β* were significantly decreased. The fractions of EtOH, EA-47, and B50M decreased the level of sAPPs to 76.73 ± 9.11%, 78.99 ± 7.02%, and 79.44 ± 8.48%, respectively. The result indicated that the fractions may inhibit the activity of *β*-secretase and then decrease the level of sAPP*β* (Figure 7), which may reflect the effects of these fractions on attenuating A*β* accumulation.

## 4. Discussion

It is generally believed that removal of A*β* from the brain might be of great benefit for AD therapy [14, 23]. To find the reagents which are capable of reducing A*β* levels is required for improving the treatment of AD. A*β* level are determined by the metabolic balance between anabolic and catabolic activities. Among the catabolic enzymes, insulin degrading enzyme (IDE) is thought to be the principal secreted enzyme responsible for the degradation of A*β* in the extracellular space [18, 21, 24]. An interesting link between insulin and A*β* is that they both are IDE substrates [20, 25, 26], and the patients with type 2 diabetes have an increased risk of AD [27]. Since IDE is more efficient on degrading insulin than A*β*, the concomitant increase in insulin and A*β* levels may lead to a redistribution of available IDE away from its function as an A*β*-degrading enzyme [25]. Thus, the involvement of IDE on A*β* degradation in our experimental system was verified by insulin and bacitracin, an IDE competitive inhibitor [28], to promote A*β* accumulation. A*β* degradation was completely abolished by 1 *μ*M Insulin, which was only partially inhibited by bacitracin. The results suggesting that IDE may be the major enzyme contribute to degrade the extracellular A*β*.

By using swAPP-N2a as cell model, we investigated the effects of *F. macrophylla *on reducing A*β* accumulation in the present of 100 nM insulin. Previous studies have indicated that some herbal medicine-derived compounds reduced A*β* accumulation in the similar cell models [29–33]. *F. macrophylla* is a popular traditional remedy used in Taiwan [1] and India [2]. The stems have been used in folk medicine for antirheumatic and anti-inflammatory agent, promoting blood circulation and antidiabetes. Our recent research has suggested the AD-relative neuroprotective effects of *F. macrophylla* on the primary cultures of neonatal cortical neurons against A*β*-mediated neurotoxicity [3]. However, the effect of *F. macrophylla* on A*β* accumulation and the underlying mechanism has not been studied. To investigate whether *F. macrophylla* affects A*β* metabolism, we detect the extracellular and intracellular A*β*1-40 levels of the treated swAPP-N2a cells by ELISA assay and found that fraction EtOH, EA-74, and B50M and flavonoid 49-3, 52-11, and 94-19-13 significantly reduced the extracellular A*β*1-40 accumulation without promoting the intracellular A*β*1-40 accumulation.

Several target sites including A*β* anabolic, trafficking, and catabolic pathways could be considered as the targets of the fractions or flavonoids on A*β* accumulation in swAPP-N2a cells. Therefore, a cell-free A*β* degradation system using N2a cell-conditioned medium as protease source and the synthetic A*β*1-40 as subtract was used to bypass the involvement of A*β* anabolic and trafficking pathway. The results showed that A*β* degradation was inhibited by insulin in a concentration-dependent manner. A*β* degradation was completely abolished by 1 *μ*M insulin. Bacitracin partially inhibited the degradation of A*β*1-40 alone or combined with insulin. It has been proposed that both microglia and astrocytes secrete protease, including IDE that mediates the degradation of A*β* in the extracellular milieu [21, 34] which may be similar to our system.

A*β* degradation by IDE was promoted by the fractions and flavonoids. In the presence of 100 nM insulin, the fractions and flavonoids decreased the remaining A*β*1-40 to about 80% of the control. The results suggest that the fractions and flavonoids may ameliorate A*β* accumulation by promoting A*β* degradation.

To study the mechanism underlying the effect of the fractions and flavonoids on the level of IDE, we first performed western blot analysis to detect IDE expression. The results showed that only flavonoid 94-18-13 significantly improved IDE expression. Nevertheless, the underlying mechanism required further investigation, although recent studies have indicated that IDE expression may be regulated through liver X receptor [35], NMDA receptor [36], *β*2 adrenergic receptor [37], insulin receptor [38], dopamine receptor [39], and glucocorticoid receptor [40].

To study the effect of the fractions and compounds on the IDE-dependent degradation pool of extracellular A*β*, we then detected extracellular A*β*1-40 levels with ELISA after treating swAPP-N2a cells with the fractions or flavonoids in the absence or presence of 2 nM bacitracin. We found that three fractions and flavonoid 94-19-13 activated IDE without affecting the bacitracin-sensitive pool, which was partially compressed and extended by flavonoid 49-3 and 52-11, respectively, through the allosteric regulatory effect. In the previous study, Cabrol et al. [41] discovered two small molecule activators of IDE through high-throughput compound screening. They established the putative ATP-binding domain as a key modulator of IDE proteolytic activity. ATP inhibits IDE-mediated insulin degradation at physiological concentration [42]. On the other hand, ATP was found to activate IDE-mediated fluorogenic substrate by conformational switch through its triphosphate moiety [43, 44]. Recently, the allosteric regulatory sites of IDE were identified [45]. Therefore, the fractions and flavonoids may activate IDE by occupying the allosteric binding site.

To determine whether the fractions affect A*β* anabolism, the western blot of medial sAPPs (sAPP*α* and sAPP*β*) was performed. The results showed that all three fractions ameliorated the production of sAPPs but not sAPP*α*, suggesting that sAPP*β* was affected by these three fractions through inhibiting the activity of *β*-secretase. The previous studies have demonstrated that the tenuigenin isolated from *Polygala tenuifolia* and berberine isolated from *Coptidis rhizome* can inhibit the secretion of A*β* via *β*-secretase inhibition [30–32]. 

## 5. Conclusion

The results suggested that the fraction EtOH, EA-74, and B50M of *F. macrophylla* may modify A*β* accumulation by both inhibiting *β*-secretase and activating IDE. The three flavonoids may modify A*β* accumulation by activating IDE. The activated IDE poor by these three flavonoids was distinctly regulated by bacitracin. Furthermore, flavonoid 94-18-13 also modulates A*β* accumulation by enhancing IDE expression. Change in A*β* accumulation may prevent A*β* aggregation and the subsequent neurotoxicity on AD. Such information could be exploited to develop the new therapeutic drugs for sporadic AD.

## Figures and Tables

**Table 1:** Extraction and isolation of *F. macrophylla.* The ground aerial parts of *F. macrophylla* (12 kg) were extracted following the protocol, and the fractions were named.

Fraction name	The protocol of extraction and fractionation
EtOH	The aerial parts of *F. macrophylla *were extracted three times with 95% ethanol (EtOH) at 60°C overnight. The combined EtOH extract was evaporated under reduced pressure.

H_2_O	EtOH extract was taken up in water as water-soluble fraction.

H25M H50M H75M H100M	The water-soluble fraction (H_2_O) was chromatogramed over Diaion HP-20 column and eluted with 25%-, 50%-, 75%-, and 100%-methanol to give four fractions: H25M, H50M, H75M, and H100M, respectively.

EA n-BuOH	The water-soluble fraction (H_2_O) was partitioned with ethyl acetate and n-butanol successively to get two fractions: EA and n-BuOH, respectively.

B25M B50M B75M B100M	The n-BuOH fraction was chromatogramed over Diaion HP-20 column and eluted with 25%-, 50%-, 75%-, and 100%-methanol to give four fractions: B25M, B50M, B75M, B100M, respectively.

EA-n	EA and n-BuOH fractions were subjected to silica gel column chromatography using a hexane-EA-methanol gradient and EA-methanol gradient, respectively. Eleven fractions were collected as EA-n (*n* = 4, 35, 52, 55, 74, 79, 85, 94, 103, 121, 165).

Flavonoids	The fractions rich in flavonoids were separated first over a silica gel column with a 25%–60% EA/hexane gradient as eluent and then over Sephadex LH-20 columns with EA or methanol to afford flavonoids.

**Table 2:** The effects of the fractions of *F. macrophylla *on the levels of extracellular and intracellular A*β*1-40. APP-transfected N2a cells were treated with the fractions of *F. macrophylla* at the STC for 20 h. The level of extracellular and intracellular A*β*1-40 was determined by ELISA. Results are means ± SD from three independent experiments. Significant differences between control and fractions-treated cells are indicated by *P < 0.05, **P < 0.01, and ***P < 0.001.

Fractions	STC (*μ*g/mL)	A*β*1-40 (% of control)	
		Extracellular	Intracellular
EtOH	10	46.18 ± 8.19***	94.92 ± 21.28
H_2_O	100	28.16 ± 7.38***	155.62 ± 13.79***
H25M	1	78.70 ± 5.21*	nd^a^
H50M	1	86.05 ± 24.57	nd
H75M	50	13.75 ± 5.56***	212.47 ± 47.25***
H100M	1	69.98 ± 13.68**	nd
BuOH	1	63.49 ± 8.09***	nd
B25M	1	66.19 ± 18.86*	nd
B50M	10	18.66 ± 2.77***	100.36 ± 16.35
B75M	50	9.40 ± 3.05***	238.75 ± 60.32***
B100M	50	64.95 ± 9.02**	nd
EA	1	124.57 ± 35.93	nd
EA-1	10	55.16 ± 6.27**	nd
EA-4	1	119.70 ± 24.55	nd
EA-35	10	71.64 ± 13.65*	nd
EA-52	1	64.57 ± 6.94*	nd
EA-55	1	54.14 ± 22.13**	nd
EA-74	1	52.65 ± 12.65**	96.21 ± 4.71
EA-79	1	107.02 ± 10.67	nd
EA-85	1	111.94 ± 26.00	nd
EA-94	1	98.63 ± 27.61	nd
EA-103	1	77.66 ± 4.73*	nd
EA-121	1	60.82 ± 18.82**	nd
EA-165	1	69.57 ± 19.16*	nd

^a^nd, not determined.

**Table 3:** The effects of the flavonoids isolated from *F. macrophylla* on the levels of extracellular and intracellular A*β*1-40. APP-transfected N2a cells were treated with the flavonoids at STC for 20 h. The level of extracellular and intracellular A*β*1-40 was determined by ELISA. Results are means ± SD from three independent experiments. Significant differences between control and flavonoid-treated cells are indicated by *P < 0.05, and **P < 0.01.

Flavonoid	STC (*μ*g/mL)	A*β*1-40 (% of control)	
		Extracellular	Intracellular
49-3	0.1	66.93 ± 11.16*	96.03 ± 5.64
52-11	0.1	65.41 ± 16.90*	99.36 ± 9.81
85-29	0.1	94.26 ± 18.15	nd
92-26	1	87.00 ± 29.13	nd
94-18-13	0.1	56.83 ± 7.52**	104.23 ± 12.40

^a^nd, not determined.
